# Typische Notfälle in der Hals-Nasen-Ohren-Heilkunde – eine monozentrische Evaluation über den jahreszeitlichen Verlauf

**DOI:** 10.1007/s00106-022-01185-7

**Published:** 2022-06-03

**Authors:** R. Lochbaum, S. Tewes, TK. Hoffmann, J. Greve, J. Hahn

**Affiliations:** Klinik für Hals-Nasen-Ohrenheilkunde, Kopf- und Halschirurgie, Universitätsklinik Ulm, Frauensteige 12, 89075 Ulm, Deutschland

**Keywords:** Akute Tonsillitis, Jahreszeit, Otitis, Nasenpyramidenfraktur, Peritonsillarabszess, Acute tonsillitis, Season, Otitis, Nasal bone fracture, Peritonsillar abscess

## Abstract

**Hintergrund:**

Die Inzidenz der Akutdiagnosen im Hals-Nasen-Ohren(HNO)-Bereich wird durch multiple Parameter beeinflusst, unter anderem meteorologische und saisonale Einflüsse. Durch ein besseres Verständnis dieser Faktoren könnten prophylaktische Ansätze entwickelt werden.

**Material und Methoden:**

Über 6 Jahre erfolgte eine retrospektive Analyse aller Patienten, die sich aufgrund von typischen und weniger komplexen HNO-ärztlichen Krankheitsbildern wie Cerumen obturans, akuter Otitis externa und media, Nasenpyramidenfraktur, Epistaxis nasi, akuter Tonsillitis, akuter Rhinosinusitis oder eines Peritonsillarabszess in einer südwestdeutschen Universitätsklinik vorgestellt haben.

**Ergebnisse:**

32.968 Fälle wurden ausgewertet. Mit 24,5 % (8082 Fälle) war die Epistaxis nasi die häufigste Notfalldiagnose. Diese, wie auch die akute Otitis media und die akute Rhinosinusitis, traten signifikant häufiger in der kälteren Jahreshälfte auf. Es bestand keine signifikante Korrelation der Diagnose Nasenpyramidenfraktur mit besonderen Zeiten wie Feiertagen. Die akute Otitis externa korrelierte signifikant mit dem Zeitraum der Sommerferien. In Kalenderwoche 38 gab es die wenigsten und in der Kalenderwoche 52 die meisten Notfallvorstellungen.

**Schlussfolgerung:**

Saisonale und meteorologische Faktoren spielen eine Rolle in der Krankheitsentstehung verschiedener HNO-ärztlicher Notfalldiagnosen.

## Abstract (en)

**Background:**

The incidence of acute ear, nose, and throat (ENT) diagnoses is influenced by multiple parameters, for example meteorological and seasonal influences. By better understanding the triggering factors, prophylactic approaches can be developed.

**Materials and methods:**

A retrospective analysis was performed over 6 years of all patients who presented to the university hospital because of typical and minor ENT emergencies. The diagnoses impacted cerumen, acute otitis media, acute otitis externa, nasal bone fracture, epistaxis, acute tonsillitis, acute rhinosinusitis, and peritonsillar abscess were analyzed.

**Results:**

A total of 32,968 cases were analyzed. Epistaxis was the most common of the investigated emergency diagnoses, accounting for 24.5% (8082 cases). Epistaxis, acute otitis media, and acute rhinosinusitis occurred significantly more frequently in the colder half of the year. There was no significant correlation of the incidence of nasal bone fracture with public holidays. Acute otitis externa correlated significantly with the local summer vacations. In calendar week 38, the fewest total emergency presentations occurred, whereas in calendar week 52, the most emergency presentations occurred.

**Conclusion:**

Seasonal and meteorological factors play a significant role in the pathogenesis of many emergency ENT diagnoses.

Akutvorstellungen in der Hals-Nasen-Ohren(HNO)-Heilkunde reichen von Erkrankungen mit geringer Dringlichkeit, wie Cerumen obturans, bis zu akut vital bedrohlichen Diagnosen, wie Luftnot und starken Blutungen. Einige auslösende Faktoren für das Auftreten von Notfallerkrankungen im HNO-Bereich sind beeinflussbar, sodass eine entsprechende Aufklärung bei Patienten und/oder zuweisenden Medizinern zu einer Reduktion von Notfallerkrankungen oder der Notwendigkeit einer Vorstellung im Notdienst führen kann.

## Hintergrund

Die Inzidenz der Akutdiagnosen im Hals-Nasen-Ohren(HNO)-Bereich wird durch multiple Parameter beeinflusst. Hierzu gehören das patientenindividuelle Verhalten, Komorbiditäten und allgemeine Umstände wie zeitliche oder lokale Risikofaktoren. Ein solcher Umstand ist die Jahreszeit, welche u. a. durch Temperaturänderung und entsprechend angepasste Freizeitaktivitäten der Patienten zum vermehrten Auftreten einzelner Diagnosen führt. Epistaxis nasi, Otitis externa und Otitis media sowie Traumata gehören zu den häufigsten Notfalldiagnosen in der HNO-Heilkunde [1, 2]. Viele der Akutdiagnosen im HNO-Bereich werden der kalten Jahreszeit zugeordnet, da beispielsweise virale und bakterielle Infekte im Bereich der oberen Atemwege nicht nur für sich eine akute ärztliche Vorstellung mit sich ziehen, sondern wiederum konsekutiv eine Otitis media oder Epistaxis nasi als Folge haben können [3, 4]. In bisherigen Studien wird auch für Abszesse im Bereich der Mundhöhle und des Pharynx ein Zusammenhang mit meteorologischen Aspekten diskutiert. Eine brasilianische retrospektive Untersuchung postulierte beispielsweise, dass Peritonsillarabszesse vermehrt in den wärmeren Monaten auftreten [5]. Ebenso fand eine deutsche Studie Hinweise für einen Zusammenhang der Inzidenz von odontogenen Abszessen und der Außentemperatur [6]. Hingegen konnten zwei deutsche retrospektive Studien keinen statistischen Zusammenhang zwischen odontogenen Abszessen und meteorologischen Parametern wie Temperatur, Atmosphärendruck und relativer Luftfeuchtigkeit finden [7, 8].

Der Einfluss einzelner Diagnosen untereinander ist ein weiterer Diskussionspunkt. Ein Beispiel ist der Zusammenhang von akuter Tonsillitis und Peritonsillarabszessen. Gegenübergestellt werden die Hypothesen der Peritonsillarabszessentstehung als Komplikation einer akuten Tonsillitis versus Abszessentstehung aufgrund von einer Blockade des Weber-Drüsen-Ausführungsgangs am oberen Tonsillenpol [9, 10].

Auch die akute virale Rhinosinusitis gehört zu den häufigsten HNO-Notfallvorstellungen mit Einfluss auf viele weitere Diagnosen im HNO-Bereich. Trotz der zunächst erscheinenden Banalität ist das Krankheitsbild nicht zu unterschätzen. Eine schwedische Fragebogenstudie mit über 1000 ausgewerteten Bögen zeigte einen substanziellen Einfluss auf die Gesellschaft durch die akute Rhinitis mit durchschnittlich 5,1 Fehldiagnosen aufgrund der Erkrankung pro Bürger pro Jahr [11]. Trotz vorwiegend leichter Krankheitsverläufe können Komplikationen in Form von intraorbitaler oder intrakranieller Infektionsausbreitung auftreten [12]. Weitere häufige Komplikationen sind die Otitis media über aufsteigende Keime durch die Eustachi’sche Röhre sowie deren Komplikationen [13]. Auch das Auftreten von Epistaxis nasi wird als mögliche Komplikation einer Infektion des oberen Atemtrakts zugeschrieben [14]. Das jahreszeitabhängig vermehrte Auftreten von Epistaxis nasi wird in vielen Studien befürwortet, dennoch gibt es auch hier einige Untersuchungen, die keinen statistisch signifikanten Zusammenhang zwischen dem Auftreten von Epistaxis nasi und meteorologischen Einflüssen finden konnten [15].

Die Diagnose akute Otitis externa hingegen ist klar assoziiert mit Wärme und hoher Luftfeuchtigkeit. Ihre Inzidenz ist in tropischen Gebieten merklich höher als in gemäßigteren Temperaturzonen. Prädisponierende Faktoren reichen vom regelmäßigen Schwimmen über dermatologische Grunderkrankungen bis zu lokalen Traumata [16, 17].

Zu den weiteren häufigen Notfalldiagnosen im HNO-Bereich gehört die Nasenpyramidenfrakur. Diese tritt signifikant häufiger bei Männern im Alter von 19–29 Jahren auf [18]. Zu den häufigsten Ursachen gehören Verkehrsunfälle, tätliche Auseinandersetzungen und Stürze, wobei die jeweilige Häufigkeit zwischen den Kontinenten und Altersgruppen differiert [19].

Die Definition eines medizinischen Notfalls ist eigentlich ein akuter, lebensbedrohlicher klinischer Zustand [20]. Da im medizinischen Alltag der Begriff „Notfall“ oft Patienten bezeichnet, die aufgrund von akuten Beschwerden eine medizinische Einrichtung außerhalb der regulären Öffnungszeiten bzw. ohne eigentlichen Termin konsultieren, wird auch in diesem Artikel die Bezeichnung „Notfall“ für die untersuchten Diagnosen verwendet. Die Fallzahlen in Notaufnahmen nehmen kontinuierlich zu. In einem Positionspapier für eine Reform der medizinischen Notfallversorgung in deutschen Notaufnahmen wird von jährlichen Steigerungen der Fallzahlen von 4–8 % gesprochen [21]. Gründe hierfür werden unter anderem in der zunehmenden Multimorbidität der Bevölkerung und der Reduktion von medizinischen Versorgungsstrukturen gesehen. Ziel unserer retrospektiven Arbeit war es mitunter, weitere allgemeine Faktoren in einer süddeutschen Universitätsklinik zu untersuchen, die zu einer erhöhten Inzidenz von einzelnen Akutdiagnosen führt. Ein Schwerpunkt war dabei der saisonale Verlauf der Notfallvorstellungen und der Zusammenhang einzelner Diagnosen untereinander.

Folgende Hypothesen zu den Notfalldiagnosen sollten in der vorliegenden Arbeit untersucht werden:

- Die Otitis externa tritt zusammenhängend mit den lokalen Sommerferien vermehrt auf.
- Notfalldiagnosen der inneren Nase und des Nasenrachensystems sowie des Mittelohrs (Otitis media, akute Rhinosinusitis und Epistaxis nasi) treten vermehrt in der kälteren Jahreshälfte auf.
- Entzündliche Notfälle der Tonsillen treten vermehrt in der kälteren Jahreszeit auf (akute Tonsillitis, Peritonsillarabszess).
- Das Auftreten der Diagnosen Peritonsillarabszess und akute Tonsillitis korreliert miteinander.
- Die Diagnose akute Nasenpyramidenfraktur tritt vermehrt in Wochen mit einem gesetzlichen Feiertag auf.

## Studiendesign und Methodik

Es wurde eine digitale Auswertung der Notfalldiagnosen in der HNO-Heilkunde durchgeführt. Die Auswertung erfolgte auf Basis der ICD(„International Statistical Classification of Diseases and Related Health Problems“)-10-Diagnosecodes mithilfe des SAP-basierten Krankenhausinformationssystems i.s.h.med® (Cerner Corporation, North Kansas City, MO, USA). Der ausgewertete Zeitraum betrug sechs Jahre (01.01.2013–31.12.2018).

Ausgewertet wurden alle im i.s.h.med® dokumentierten ambulanten und stationär aufgenommenen Fälle mit den im folgenden genannten Diagnosen. Bewusst wurden häufige Notfalldiagnosen ausgewählt, die hinsichtlich der genannten Hypothesen und in Abhängigkeit zum saisonalen Verlauf in der Auswertung von Interesse waren und zudem rein aufgrund des klinischen Bildes richtig diagnostiziert werden – ohne weitere Gerätediagnostik, die im Notdienst nicht immer verfügbar ist. Die Auswertung der Häufigkeit ist dementsprechend nur untereinander und anhand der Absolutzahlen möglich, nicht im allgemeinen Vergleich zur Analyse aller Notfalldiagnosen in einer HNO-Klinik. Es handelt sich um die Auswertung eines Universitätsklinikums im süddeutschen Raum mit einem Einzugsgebiet von etwa 100 km Radius.

Die folgenden Diagnosen wurden berücksichtigt:

- H61.2 Cerumen
- H60.3 Otitis externa
- H66.0 Akute Otitis media
- J03. Akute Tonsillitis
- J63 Peritonsillarabszess
- R04.0 Epistaxis nasi
- J01. Akute Sinusitis
- S02.2 Nasenpyramidenfraktur

Ausgewertet wurde die jeweilig erfasste Hauptdiagnose, die im Rahmen der Patientenvorstellung ermittelt wurde. Es erfolgte eine primär deskriptive Analyse. Die Vorstellungen wurden in die einzelnen Kalenderwochen im Jahr eingeteilt. Summe, Median und Mittelwert wurden ermittelt. Mittels Microsoft Excel und GraphPad Prism wurden graphische Darstellungen erstellt.

Die statistische Analyse wurde mit GraphPad Prism durchgeführt. Es erfolgten Korrelationsanalysen zu zeitlichen Faktoren wie Ferien bzw. Feiertagen. Diese wurden mittels der nichtparametrischen Korrelationsanalyse nach Spearman untersucht. Signifikanzanalysen wurden mit dem zweiseitigen Mann-Whitney-U-Test durchgeführt. Generell wurde bei einem *p*-Wert von ≤ 0,05 ein statistisch signifikanter Zusammenhang angenommen.

## Ergebnisse

**Figure 1:**
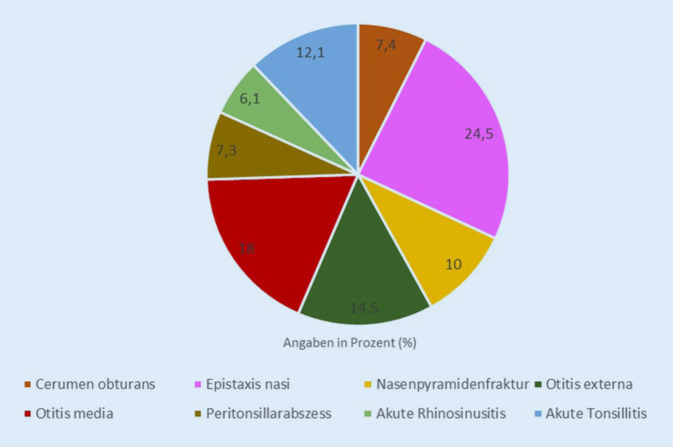

Insgesamt wurden 32.968 Fälle ausgewertet. Im Folgenden sind die Ergebnisse nach Häufigkeit in Absolutzahlen aufgelistet. Die Abb. 1 stellt den prozentualen Anteil dar.

1. Epistaxis nasi (8082 Fälle)
2. Otitis media (5918 Fälle)
3. Otitis externa (4781 Fälle)
4. Akute Tonsillitis (3998 Fälle)
5. Nasenpyramidenfraktur (3313 Fälle)
6. Cerumen obturans (2451 Fälle)
7. Peritonsillarabszess (2411 Fälle)
8. Akute Rhinosinusitis (2014 Fälle)

**Figure 2:**
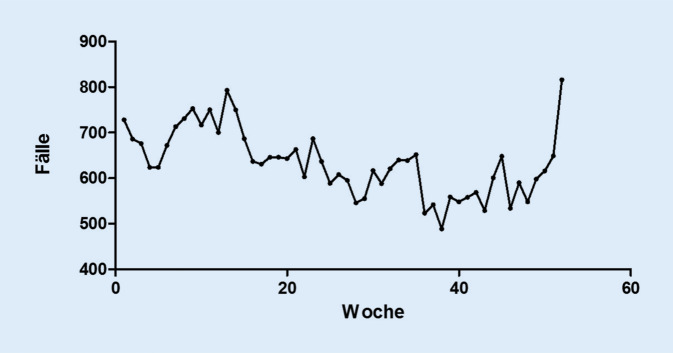

Alle Fälle wurden als Absolutzahlen summiert und den jeweiligen Kalenderwochen zugeordnet. Hierdurch wurde ein Verlauf der Fälle über das Jahr herausgearbeitet (Abb. 2). In Kalenderwoche 38 waren in der Summe die wenigsten Notfallvorstellungen aufgrund der genannten Diagnosen, in Kalenderwoche 52 kam es summiert zu den meisten Notfallvorstellungen.

### Notfälle äußeres Ohr

**Figure 3:**
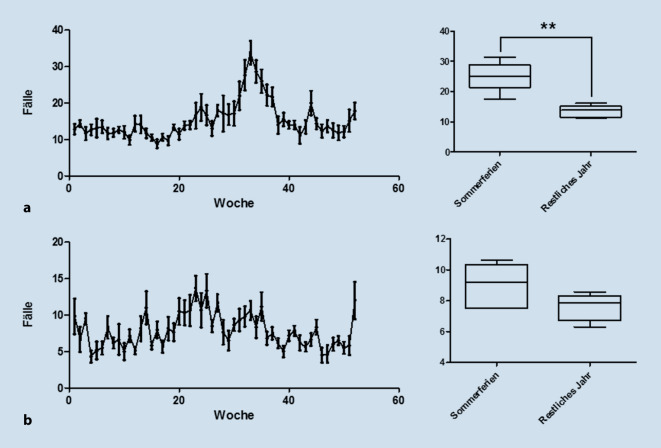

Die Diagnosen Cerumen obturans und Otitis externa wurden ebenfalls im Jahresverlauf nach Kalenderwochen analysiert. Hier zeigte sich für die Otitis externa eine deutliche Fallzunahme in den Sommermonaten. Es wurde eine Signifikanztestung mittels zweiseitigem Mann-Whitney-U-Test durchgeführt, wobei ein Zusammenhang zwischen dem Auftreten der Diagnosen und den Sommerferien (Bayern und Baden-Württemberg) gegenüber den restlichen Wochen im Jahr untersucht wurde. Hier zeigte sich ein signifikanter Zusammenhang zwischen dem Auftreten der Otitis externa und den Sommerferien (*p* < 0,01; Abb. 3a) und kein signifikanter Zusammenhang zwischen dem Auftreten von Cerumen obturans und den Sommerferien. (*p* = 0,1986; Abb. 3b).

### Notfälle innere Nase, Nasenrachensystem und Mittelohr

**Figure 4:**
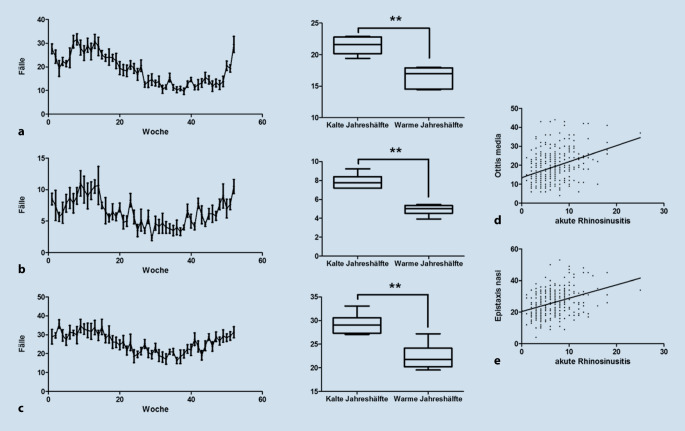

Die Diagnosen Epistaxis nasi, akute Rhinosinusitis und die akute Otitis media wurden als Notfalldiagnosen der inneren Nase und des Nasenrachensystems ausgewertet. Neben der summierten Auswertung über die Kalenderwochen des Jahres erfolgte eine Signifikanztestung hinsichtlich des Auftretens in der kälteren und wärmeren Jahreszeit. Hierzu wurden die Kalenderwochen 1–14 sowie 41–52 als kältere Jahreszeit und die Kalenderwochen 15–40 als warme Jahreszeit definiert. Hier zeigte sich bei allen drei Diagnosen ein signifikanter Zusammenhang zur kalten Jahreshälfte (Otitis media: *p* = 0,0022; Abb. 4a; akute Rhinosinusitis: *p* = 0,005; Abb. 4b; Epistaxis nasi: *p* = 0,0043; Abb. 4c; Mann-Whitney-U-Test).

### Notfälle des Oropharynx

**Figure 5:**
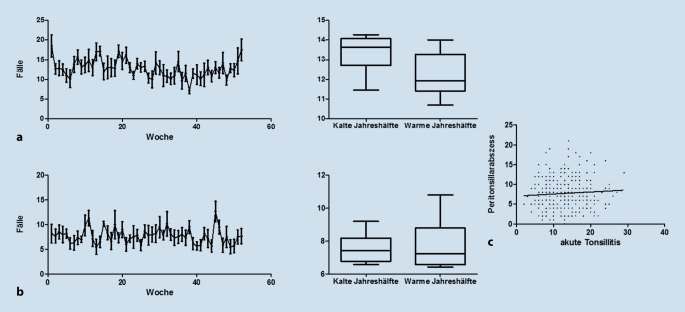

Zu den Notfällen im Bereich der Tonsillen wurde die akute Tonsillitis und der Peritonsillarabszess ausgewertet. Auch hier erfolgte neben den Absolutwerten im zeitlichen Verlauf über das Jahr eine Signifikanztestung hinsichtlich des Auftretens in der kälteren und wärmeren Jahreszeit (Abb. 5). Hierzu wurden wie im eben genannten Abschnitt die Kalenderwochen 1–14 sowie 41–52 als kältere Jahreszeit und die Kalenderwochen 15–40 als warme Jahreszeit definiert. Es zeigte sich bei beiden Diagnosen kein signifikanter Zusammenhang (akute Tonsillitis: *p* = 0,1488; Peritonsillarabszess: *p* = 1,00; Mann-Whitney-U-Test).

Darüber hinaus wurde eine Korrelationsanalyse durchgeführt, ob das zeitliche Auftreten der Diagnose akute Tonsillitis mit dem Auftreten der Diagnose Peritonsillarabszess korreliert. Auch hier ergab sich in der nichtparametrischen Korrelationsanalyse nach Spearman (r_s_ = 0,05536) kein Anhalt für eine Korrelation (*p* = 0,3927).

### Notfall Nasenpyramidenfraktur

**Figure 6:**
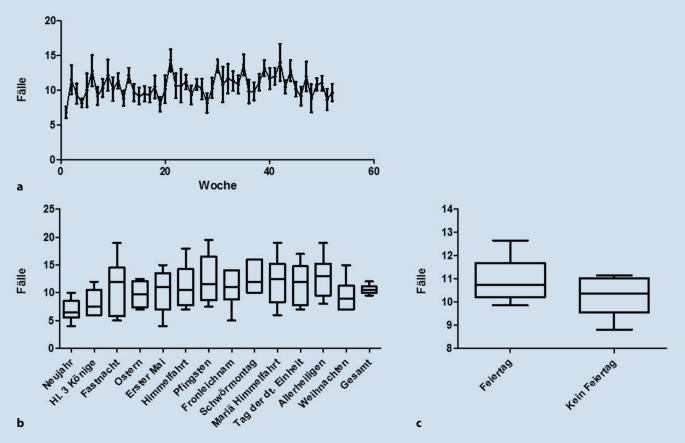

Die Notfalldiagnose Nasenpyramidenfraktur wurde in Absolutzahlen über den Jahresverlauf ausgewertet (Abb. 6a). Zur Analyse der Risikofaktoren erfolgte zusätzlich eine Auswertung der Häufigkeit des Auftretens der Notfalldiagnose Nasenpyramidenfraktur in der Woche eines gesetzlichen Feiertags. Dabei wurden die Feiertage in den süddeutschen Bundesländern berücksichtigt. In der Untersuchung konnte kein signifikanter Zusammenhang festgestellt werden (*p* = 0,4704; Mann-Whitney-U-Test, Abb. 6c).

## Diskussion

In der retrospektiven Studie einer Universitätsklinik zu akuten Notfalldiagnosen im HNO-Bereich mit insgesamt 32.968 Fällen wurden fünf zuvor aufgestellte Hypothesen bearbeitet und für die einzelnen Diagnosen eine deskriptive Auswertung hinsichtlich ihres Auftretens im Jahresverlauf durchgeführt. Mit 24,5 % (8082 Fälle) war die Epistaxis nasi in der vorliegenden Studie die häufigste Notfalldiagnose. Dies entspricht dem Ergebnis der retrospektiven Untersuchung von Kuhr et al., in welcher die Epistaxis nasi ebenfalls die am häufigsten aufgetretene HNO-Notfalldiagnose war [20]. Bolz et al. führten 2004 eine retrospektive Auswertung in einer HNO-Notfallambulanz der Universitätsklinik Köln durch. Hier waren die Otitis media gefolgt von der Otitis externa die häufigsten Notfalldiagnosen [22]. Mitunter einen Einfluss auf die Häufigkeit der einzelnen Diagnosen nimmt sowohl das Einzugsgebiet als auch die Größe der Stadt und das Vorhandensein weiterer Anlaufstellen, wie HNO-Notdienste der kassenärztlichen Vereinigung (KV). Somit ist an dieser Stelle als erste Limitation die unizentrische Auswertung der Studie zu nennen, die im Fall der vorliegenden Studie eine HNO-Universitätsklinik ohne naheliegende weitere Universitätsklinik mit einem Einzugsgebiet von ungefähr 100 km Radius umfasst. Dies sind Faktoren, die relevanten Einfluss auf die Notfallvorstellungen haben und im Fall der vorliegenden Klinik auch zeigen, wie hoch die Anzahl der Vorstellung von Bagatelldiagnosen ist, die in einer fachspezifischen Universitätsklinik entsprechende Ressourcen binden.

Auch das Behandlungsspektrum mitinvolvierter Disziplinen wie der Pädiatrie oder der Mund-Kiefer-Gesichts-Chirurgie beeinflusst das Notdienst-Patientenkollektiv relevant und stellt aufgrund der schwierigen Berücksichtigung in einer solchen unizentrischen Auswertung eine Limitation dar [22].

Im Folgenden sollen die Ergebnisse zu den im Vorfeld aufgestellten Hypothesen diskutiert werden.

Die Inzidenz der Otitis externa zeigte sich in der vorliegenden Studie signifikant assoziiert mit dem Zeitraum der lokalen Sommerferien. Dies unterstützt die Annahme, dass das individuelle Verhalten wie vermehrtes Baden und Schwimmen einen relevanten Einfluss auf die Inzidenz der Erkrankung hat. Zumeist handelt es sich bei der Otitis externa um ein unkompliziertes Krankheitsbild, zur generellen Prognose ist es schwierig, valide Daten zu finden [23]. Da eine feuchte Umgebung bekanntermaßen ein Risikofaktor für die Otitis externa ist, könnten einfache Maßnahmen wie das konsequente Trockenföhnen der Ohren nach dem Baden sowie eine generelle Gehörgangspflege insbesondere bei Patienten mit weiteren Risikofaktoren (z. B. Hörgeräteträger) die Inzidenz der Otitis externa reduzieren und somit die Notaufnahmen entlasten. Basis wäre hierfür eine Patientenaufklärung im Alltag, vorwiegend durch HNO-ärztliche, pädiatrische oder allgemeinärztliche Praxen.

Über den Nasopharynx besteht eine enge anatomische und funktionelle Verbindung infektiöser Notfalldiagnosen wie der Otitis media und der akuten Rhinosinusitis, was die positive Korrelation in unserer Auswertung zwischen beiden Erkrankungen erklärt. Zur Verbesserung der Belüftung des Mittelohrs bei akuter Rhinosinusitis – und somit zur Prävention einer akuten Otitis media als Komplikation – sowie zur Verhinderung eines komplizierten Verlaufs einer Sinusitis wäre die temporäre Anwendung von topischen Dekongestiva eine leicht durchführbare Therapieoption. Die Datenlage hierzu ist bislang trotz der Häufigkeit der Krankheitsbilder unzureichend [24]. Die positive Korrelation unserer Auswertung zwischen Epistaxis nasi und akuter Rhinosinusitis hingegen kann auf unterschiedlichen Wegen diskutiert werden. Zum einen bestehen bei beiden Erkrankungen ähnliche Risikofaktoren, wie z. B. eine trockene Umgebungsluft und digitale Manipulation. Zum anderen führt eine alterierte Schleimhaut im Rahmen einer Rhinosinusitis zu einer vermehrten Blutungstendenz, was einen kausativen Zusammenhang annehmen lässt. Auch hier bestehen einfach durchzuführende Präventionsmaßnahmen in konsequenter Nasenpflege und der Anwendung von schleimhautbefeuchtenden Maßnahmen.

Zunächst unerwartet in der vorliegenden Auswertung war das Ergebnis, dass die Diagnose akute Tonsillitis keine signifikante Korrelation zur Saison aufwies, was entgegen der Aussage einiger bisher vorliegender Daten ist [25]. Eine aktuelle chinesische Studie fand sogar eine Assoziation des Auftretens der akuten Tonsillitis zu höheren Temperaturen [26]. Hinsichtlich der Assoziation der akuten Tonsillitis zum Peritonsillarabszess wurde initial angenommen, dass ein Peritonsillarabszess aus einer akuten Tonsillitis entsteht. Das vorliegende Ergebnis, dass das Auftreten der akuten Tonsillitis nicht mit dem Auftreten der Diagnose „akuter Peritonsillarabszess“ korreliert, widerspricht dieser Annahme und deutet auf die Theorie der Abszessentstehung anderer Genese, wie aus den extratonsillär gelegenen Weber-Drüsen [25]. Weiterhin bleibt diese Thematik viel diskutiert, beispielsweise stellt die Publikation von Klug et al. Argumentationen beider Seiten gegenüber [10].

Ein Bruch der Nasenpyramide ist die häufigste Fraktur im Bereich des Gesichts [27]. Insbesondere isolierte Nasenpyramidenfrakturen treten vorwiegend durch menschliche Auseinandersetzungen auf [28]. Die formulierte Hypothese, dass beispielsweise durch vermehrten Alkoholkonsum in Wochen von gesetzlichen Feiertagen die Inzidenz von Nasenpyramidenfrakturen steigt, konnte in der vorliegenden Studie nicht bestätigt werden.

Da die Studie vor der aktuellen Coronaviruspandemie durchgeführt wurde, sind die Zahlen nicht durch das Tragen des Mund-Nasen-Schutzes oder andere pandemiespezifische Faktoren beeinflusst. In vielen nationalen und internationalen Studien wurde inzwischen gezeigt, dass während der Coronapandemie die Notfallvorstellungen aus diversen Gründen zurückgingen. Die häufigsten Diagnosen vor und während der COVID-19-Pandemie hatten sich in einer Untersuchung einer HNO-Klinik aus Süddeutschland jedoch nicht signifikant verändert. Epistaxis nasi war weiterhin die häufigste Ursache für eine Akutvorstellung im HNO-Notdienst während der Coronapandemie, wenn auch mit geringeren Absolutzahlen [29].

Die schon zitierte retrospektive Untersuchung von Kuhr et al., in der auch die zuweisende Quelle der Notfallvorstellungen in einer deutschen HNO-Universitätsklinik untersucht wurde, zeigte, dass Notfallpatienten vorwiegend aufgrund von Eigeninitiative vorstellig wurden. Zweithäufigste Fälle waren Überweisungen durch Allgemeinmediziner, und an dritter Stelle standen Überweisungen durch HNO-Fachärzte [20]. Hier zeigt sich, dass Informationen zur potenziellen Prävention von HNO-Erkrankungen und einfach durchführbare Eigenmaßnahmen im Fall von Symptomen vorwiegend an Patienten selbst bzw. Allgemeinmediziner adressiert werden sollten.

## Limitationen

Eine Limitation der vorliegenden Studie ist das retrospektive Setting, welches hingegen die große Patientenzahl ermöglichte. Eine weitere Limitation sind unbekannte Faktoren, die auf die vorliegenden Zahlen Einfluss nahmen, ohne dass sie quantitativ erfasst werden konnten. Hierzu gehören beispielsweise die Öffnungszeiten und Besetzung umliegender HNO-Praxen, die zu einem generellen Anstieg der Absolutzahlen der Notfallpatienten in der Universitätsklinik an Feiertagen und an Wochenenden führen kann. Zudem wurden in der vorliegenden Arbeit ausgewählte Diagnosen ausgewertet. Spezielle Charakteristika, wie Alter und Geschlecht der Patienten, sowie individuelle Risikofaktoren oder Verläufe waren nicht Teil der Auswertung. Zudem wurde nur die aktuelle Hauptdiagnose erfasst. Es ist somit möglich, dass bei Patienten, bei denen eine akute Rhinosinusitis diagnostiziert wurde, parallel eine leichte Otitis media vorlag, die keinen Einfluss auf die Auswertung hatte. Berücksichtigt werden muss auch, dass die Analyse vor der aktuellen Coronaviruspandemie durchgeführt wurde. Somit bestand kein Einfluss auf die Daten durch die zwischenzeitlich eingeführte Maskenpflicht und/oder Quarantäneregelungen.

## Ausblick

Die Patientenfallzahlen in Notaufnahmen nehmen kontinuierlich zu, und Personalprobleme in medizinischen Einrichtungen sind ein mehr denn je diskutiertes Thema. Die vorliegenden Zahlen verdeutlichen, dass der HNO-Notdienst insbesondere in der kälteren Jahreszeit frequentiert wird. Hauptursache liegt in der höheren Inzidenz einiger der häufigsten Notfalldiagnosen im HNO-Bereich, wie der Epistaxis nasi, akuten Otitis media und akuten Rhinosinusitis. Diskutiert werden kann in diesem Zusammenhang eine optimierte Personalbesetzung in der kälteren Jahreshälfte. Prophylaktische Maßnahmen könnten die Inzidenz der meisten Notfallvorstellungen zudem reduzieren und somit die Belastung der Notaufnahmen verringern. Eine Verringerung der Inzidenz der akuten Otitis externa könnte durch eine bessere Patientenaufklärung erreicht werden, welche das bestmögliche Trockenhalten der äußeren Gehörgänge insbesondere im Sommerurlaub beinhaltet. Das Auseinandersetzen mit leicht vermeidbaren prädisponierenden Faktoren für einzelne Notfalldiagnosen stellt einen relevanten Ansatzpunkt für die Reduktion der Patientenfallzahlen in Notaufnahmen dar.

## Fazit für die Praxis

- Der HNO-ärztliche Notdienst wird vergleichsweise häufiger in der kälteren Jahreszeit frequentiert, was durch eine signifikante Zunahme der Inzidenzen der Notfalldiagnosen im Bereich der inneren Nase und des Nasenrachensystems begünstigt wird.
- Es besteht keine signifikante Korrelation zwischen saisonalen Faktoren und den Diagnosen akuter Tonsillitis und Peritonsillarabszess sowie zwischen den beiden Diagnosen untereinander.
- Ein deutlicher Zusammenhang zwischen akuter Rhinosinusitis und akuter Otitis media bzw. Epistaxis nasi konnte gezeigt werden.
